# Salivary Biomarkers: Future Approaches for Early Diagnosis of Neurodegenerative Diseases

**DOI:** 10.3390/brainsci10040245

**Published:** 2020-04-21

**Authors:** Giovanni Schepici, Serena Silvestro, Oriana Trubiani, Placido Bramanti, Emanuela Mazzon

**Affiliations:** 1 IRCCS Centro Neurolesi “Bonino-Pulejo”, Via Provinciale Palermo, Contrada Casazza, 98124 Messina, Italy; giovanni.schepici@irccsme.it (G.S.); serena.silvestro@irccsme.it (S.S.); placido.bramanti@irccsme.it (P.B.); 2 Department of Medical, Oral and Biotechnological Sciences, University “G. d’Annunzio” Chieti-Pescara, 66100 Chieti, Italy; trubiani@unich.it

**Keywords:** salivary biomarkers, Alzheimer’s disease, Parkinson’s disease, amyotrophic lateral sclerosis, multiple sclerosis

## Abstract

Many neurological diseases are characterized by progressive neuronal degeneration. Early diagnosis and new markers are necessary for prompt therapeutic intervention. Several studies have aimed to identify biomarkers in different biological liquids. Furthermore, it is being considered whether saliva could be a potential biological sample for the investigation of neurodegenerative diseases. This work aims to provide an overview of the literature concerning biomarkers identified in saliva for the diagnosis of neurodegenerative diseases such as Alzheimer’s disease (AD), Parkinson’s disease (PD), amyotrophic lateral sclerosis (ALS), and multiple sclerosis (MS). Specifically, the studies have revealed that is possible to quantify beta-amyloid_1–42_ and TAU protein from the saliva of AD patients. Instead, alpha-synuclein and protein deglycase (DJ-1) have been identified as new potential salivary biomarkers for the diagnosis of PD. Nevertheless, future studies will be needed to validate these salivary biomarkers in the diagnosis of neurological diseases.

## 1. Introduction

Neurodegenerative diseases show a progressive neuronal degeneration that induces motor and cognitive deficits. Molecular mechanisms such as excitotoxicity, intracellular inclusions, and the extracellular aggregation of toxic molecules, as well as mitochondrial dysfunction, can induce neuronal degeneration [1]. Therefore, the identification of biomarkers in biological fluids is important to carry out the diagnosis and evaluation of the clinical state of the disease [1]. Cerebrospinal fluid (CSF), being directly linked with the central and peripheral nervous tissue, contains specific markers involved in the pathophysiological state of neurological disorders. Moreover, CSF is the most used material for the study of biomarkers in neurodegenerative diseases. However, CSF sampling is a very invasive procedure, as it requires a lumbar puncture.

Saliva is an interesting biomaterial composed of serous and mucous secretions containing alpha-amylase, mucin and ions [2]. Saliva is a biological fluid that exerts its digestive properties through the secretion of enzymes. Moreover, thanks to its lubricating and antibacterial properties, saliva protects both oral tissues and teeth [3]. Saliva is secreted in the mouth by different salivary glands such as those of the submandibular, parathyroid, and sublingual. The facial nerve innervates the sublingual and submandibular glands [4], whereas the glossopharyngeal nerve innervates the parathyroid gland [1]. Therefore, considering the direct relationship between the nervous system and the salivary glands, attention has been focused on the use of saliva as a fluid potential to detect the biomarkers of neurodegenerative diseases [5].

Several techniques have been used for saliva sampling. Unstimulated saliva can be directly collected in a plastic tube from the burr. This method, known as passive collection, has the advantage that most analytes can be quantified without problems. By contrast, it has the disadvantage of having a low volume of sample. Moreover, salivation can be induced through gustatory or masticatory stimulations or by the use of citric acid. However, sampling in patients with very advanced neurodegenerative diseases is very complex. Indeed, the saliva sample is collected by the cannulation of the glandular ducts or by using appropriate devices positioned where the glandular ducts protrude. The stimulation of saliva allows for the obtainment of a larger quantity of sample. Likewise, it is a complex method that requires attention for its collection [6,7].

After collection, salivary samples are centrifuged and the supernatants are frozen for subsequent analysis. The most used methods for the determination of salivary biomarkers are the enzyme-linked immunosorbent assay (ELISA), multiplex array assays, the ultra-sensitive single-molecule array, the immunoassay with magnet nanoparticles, and Western blot. 

The purpose of this manuscript was to provide an overview of clinical studies that have used saliva in order to detect biomarkers for the diagnosis of neurodegenerative diseases. In order to write this review, we carried out our research on PubMed using the following keywords "salivary biomarkers" and "neurodegenerative diseases." This research led us to find 51 studies. We describe clinical studies that have assessed the salivary quantification of new biomarkers and of those already in use in clinical practice.

## 2. Alzheimer’s Disease

Alzheimer’s disease (AD) is a neurodegenerative disease characterized by accumulations of the β amyloid peptide (Aβ), as well as neurofibrillary tangles (NFTs) [8]. Several studies have demonstrated that Aβ accumulations trigger a cascade of pathological events that induce neuronal damage [9]. It has been shown that Aβ peptide originates from the enzyme proteolysis of the amyloid precursor protein (APP), which plays an important role in brain homeostasis [10]. In physiological conditions, the APP is cleaved by the α-secretase into a soluble molecule that shows neuroprotective action [11]. Instead, in pathological conditions, the APP is processed by β and γ-secretase. The APP is cleaved by β-secretase, which generates a fragment of 99 amino acids [12]. Subsequently, the γ-secretase makes a second cut by dividing the fragment into a peptide of 40 amino acids and a peptide of 42, called, respectively, Aβ_1–40_ and Aβ_1–42_ [13]. In the extracellular space, these peptides tend to aggregate in insoluble oligomers and fibrils. The fibrils associate, forming plaques known as amyloid plaques. These plaques trigger a reactive inflammatory process that leads to neuronal damage [14].

TAU is another protein involved in AD. This protein stabilizes microtubules that are important for ensuring axonal transport and for maintaining neuronal structure and plasticity. In physiological conditions, a perfect balance between phosphorylation and dephosphorylation regulates the correct functioning of TAU. Contrarily, AD is characterized by excessive phosphorylation of TAU which causes NTF formation followed by destabilization and neuronal death [15,16].

AD’s diagnosis is performed by magnetic resonance imaging (MRI) and positron emission tomography (PET). MRI allows one to evaluate the size of the hippocampus, which is smaller in AD patients than in healthy subjects [17]. PET that uses tracers for Aβ, instead, allows for the identification of Aβ deposits in the brain [18]. Another diagnostic method used is CSF sampling through a lumbar puncture in order to analyze some markers such as Aβ_1–42_, total-TAU (t-TAU), and phosphorylated-TAU (p-TAU) [19,20]. Generally, in the CSF of patients with AD, compared to healthy patients, Aβ is reduced because it is deposited in the brain, while the two forms of TAU are increased [20,21].

### Salivary Biomarkers in Alzheimer’s Disease

Several studies have investigated the potential use of saliva to quantify markers of AD such as Aβ_1–42_, Aβ_1–40_, and TAU [22].

Bermejo-Pareja et al. quantified the levels of Aβ_1–42_ and Aβ_1–40_ in the saliva and plasma samples of 70 AD patients, 51 Parkinson’s disease (PD) patients, and 56 healthy control (HC) subjects using an ELISA kit. The authors reported a higher level of Aβ_1–42_ in the saliva of AD patients (6.81 ± 20.04 pg/mL) compared to the PD (3.66 ± 4.21 pg/mL) and HC (2.89 ± 4.96 pg/mL) subjects; the Aβ_1–40_ level in saliva was not significant in AD patients compared to HC subjects. Instead, plasma Aβ_1–42_ and Aβ_1–40_ levels did not differ significantly between the AD and HC subjects. Additionally, the study assessed the correlation between Aβ_1–42_ levels and the severity of AD. The results showed a significant increase in salivary Aβ_1–42_ level in patients with mild AD (7.67 ± 16.25 pg/mL) and patients with moderate AD (11.70 ± 34.76 pg/mL) compared to patients with severe AD (3.03 ± 3.49 pg/mL) and healthy patients (2.89 ± 4.96 pg/mL). The authors showed that, unlike in CSF, Aβ_1–42_ levels increase in saliva [23].

Kim et al., using the antibody-based magnet nanoparticles immunoassay, evaluated the salivary Aβ_1–42_ and Aβ_1–40_ levels in 28 AD patients with severe and mild cognitive impairment (MCI) and 17 HC subjects. This quantification system detected minimum concentrations (∼20 pg/mL) of the Aβ peptides from the salivary samples of AD patients. In this way, it was possible to correlate the levels of salivary Aβ with the severity of AD. The results of the study showed a significant increase in the Aβ_1–42_ level in patients with severe AD compared to HC subjects. Similarly, an increase in the salivary Aβ_1–42_ level in patients with severe AD compared to MCI patients was shown. Conversely, in AD patients, compared to HC subjects, salivary Aβ_1–40_ levels remained unchanged. These results were compared using a sensitive ELISA. Compared to the ELISA results, an antibody-based magnet nanoparticles immunoassay showed a high precision rate for the identification of Aβ_1–42_ peptides in human saliva. In conclusion, unlike the previous study, a positive correlation between the concentration of Aβ_1–42_ and disease severity was demonstrated [24].

Lee et al. quantified the Aβ_1–42_ levels in saliva and tissue samples (small intestine, kidney, pancreas, spleen, hippocampus, and sensory cortex of the brain) in 10 patients with severe AD and 27 HC using an ELISA kit. Compared to HC subjects, all organs collected from AD patients showed significant Aβ_1–42_ levels. Specifically, higher levels of Aβ_1–42_ were found in the kidney (122.6 pg/g), pancreas (128.2 pg/g), and spleen (134.3 pg/g). Instead, the intestine was the organ with lower levels of Aβ_1–42_ (80.58 pg/g), followed by the hippocampus (102.4 pg/g) and the sensory cortex of the brain (97.8 pg/g). In the salivary samples, the Aβ_1–42_ level was higher in the AD patients (59.07 ± 6.33 pg/mL) compared to the HC subjects (22.06 ± 0.41 pg/mL). However, the Aβ_1–42_ level in saliva was lesser compared to other organs. In conclusion, the results of this study showed that by using an ELISA kit, it is possible to detect the presence of Aβ_1–42_ in both peripheral organisms and in saliva [22].

Based on these findings, Sabbagh et al. quantified the salivary Aβ_1–42_ levels in 15 AD patients and 8 HC subjects. In line with previous results, the authors, using an ELISA kit, reported a significant increase in the Aβ_1–42_ levels in AD patients (51.7 ± 1.6 pg/mL) compared to HC subjects (21.1 ± 0.3 pg/mL) [25].

In addition to dosing Aβ levels in saliva, other authors have also quantified t-TAU and p-TAU levels [26]. Shi et al., by using mass spectrometry, assessed the possibility of quantifying the t-TAU, p-TAU and Aβ_1–42_ levels with this approach in saliva samples of 21 AD patients and 38 HC subjects. The results showed that this technique does not allow for the quantification of the salivary levels of Aβ_1–42_. Instead, a significant increase of the t-TAU/p-TAU ratio in AD patients (*p*-value <0.05) compared to HC subjects was reported. This result proved that the TAU protein is unequivocally present in human saliva [26]. Furthermore, these data showed that in salivary samples, as in CSF, it is possible to quantify the t-TAU/p-TAU ratio.

Lau et al. evaluated the t-TAU, p-TAU, and Aβ_1–42_ levels in the saliva samples of 20 AD patients, 20 PD patients, and 20 HC subjects using an ELISA test. The results showed that no quantitative differences in t-TAU and p-TAU were found in AD patients compared to HC subjects. However, a slight increase of p-TAU levels was observed in AD patients (*p*-value <0.05) compared to PD patients and HC subjects. Additionally, in this study, the Aβ_1–42_ biomarker was not sensed in the analyzed saliva samples. Therefore, these results are in line with data from the previous study [27].

Pekeles et al. quantified the t-TAU/p-TAU ratio on different phosphorylation sites (T181, S396 e S404, S400, T403, and T404) in saliva samples of 46 AD patients, 55 MCI patients, and 47 elderly healthy subjects by using Western blot analysis. The authors showed a significant increase in the t-TAU/p-TAU ratio (*p*-value <0.05), specifically at certain phosphorylation sites (particularly S396) in AD patients compared to MCI and elderly healthy subjects. Conversely, in the CSF of AD and elderly healthy subjects, no significant differences in p-TAU/t-TAU levels were observed. Therefore, this study showed that the p-TAU/t-TAU ratio obtained from CSF did not correlate with the values of salivary samples. As a consequence, further studies are needed to validate the salivary t-TAU/p-TAU ratio as a potential biomarker for AD [28].

Ashton et al., using the ultra-sensitive single-molecule array technology, evaluated the salivary t-TAU levels in 53 AD patients, 68 MCI patients, and 160 elderly healthy subjects. The results proved no significant difference t-TAU level in AD patients (12.3 ng/L) compared to both MCI (9.8 ng/L) and elderly healthy subjects (9.6 ng/L) [29].

In conclusion, the results of these studies showed that ELISA tests allow for the detection of Aβ_1–42_ levels in saliva. In addition, the presence of Aβ_1–42_ in saliva shows a direct correlation with disease severity. Using different techniques, it was also possible to quantify the levels of t-TAU and p-TAU in saliva. However, no correlation between the salivary p-TAU/t-TAU levels and the pathological score of AD was found.

In Table 1, we summarize all the potential salivary biomarkers correlated with AD, studied in the above mentioned clinical studies.

## 3. Parkinson’s Disease

PD is a neurodegenerative condition characterized by the progressive loss of dopaminergic neurons of the substantia nigra with a consequent reduction in the dopamine levels in the corpus striatum [30]. Moreover, in the brains of PD patients, the surviving cells contain characteristic cytoplasmic inclusions of alpha-synuclein (α-synuclein) known as Lewy bodies [31]. PD is clinically characterized by tremors, muscle stiffness, postural instability, akinesia, and bradykinesia, which occur after the loss of at least 60% in dopaminergic neurons. In addition, PD patients also present non-motor deficits such as anxiety, depression, sleep disturbances, dementia, and psychosis [32]. PD is diagnosed by clinical evaluation using the Unified Parkinson’s Disease Rating Scale (UPDRS). The UPDRS is a neurological scale used to evaluate the mental and physical conditions.

In 90% of cases, PD occurs in idiopathic form, while in 10% of patients, it can be acquired in familial form. Familial PD is characterized by autosomal dominant and recessive mutations in several genes such as α-synuclein (*SNCA*), ubiquitin C-terminal hydrolase L1 (*UCHL-1*), phosphatase and tensin homolog-induced putative kinase 1 (*PINK1*), PARKIN (*PRKN*), protein deglycase (*DJ-1)*, and leucine-rich repeat kinase 2 (*LRRK2*) [33]. Currently, clinical diagnosis for familial PD is performed by genetic sequencing. There are no biomarkers validate for the diagnosis of idiopathic PD, and the diagnosis is performed using single-photon emission computed tomography (SPECT) with the radiotracer imaging of dopaminergic transporter (DAT) and brain PET [34].

### 3.1. Salivary Biomarkers in Parkinson’s Disease

Currently, the search for biomarkers for the early diagnosis of PD is of interest to researchers. Several studies have reported interesting results regarding the quantification of α-synuclein and DJ-1 proteins in saliva, thus proposing them as potential biomarkers for PD [35].

The α-synuclein is a soluble, acidic, and heat-resistant protein that aggregates into Lewy bodies. In the CSF of PD patients, total α-synuclein decreases, showing a high predictive value in the diagnosis of PD [36]. Instead, PD patients show elevated α-synuclein levels in the blood, especially in red blood cells [37]. However, the high fragility of red blood cells could be the cause of the possible contamination of CSF [38]. Therefore, in order to resolve this problem, the quantification of α-synuclein in saliva could be a valid method for the diagnosis of PD.

Al-Nimer et al., using an ELISA kit, quantified the total α-synuclein levels in saliva samples of 20 PD patients and 20 HC subjects. The authors reported a lower level of total α-synuclein in PD patients (65 ± 52.2 pg/mL) compared to HC subjects (314.01 ± 435.9 pg/mL). These results suggested that the quantification of total α-synuclein in saliva could be used as a biomarker in the diagnosis of PD [39].

However, in healthy subjects, α-synuclein is present in the monomeric form but predominates presents as the oligomeric form in PD patients. Subsequently, the α-synuclein oligomers are converted into amyloid fibrils with the consequent formation of Lewy bodies. Vivacqua et al., using an ELISA assay, quantified the oligomeric α-synuclein in the saliva of 60 PD patients and 40 HC subjects. A decrease in the total α-synuclein in PD patients (5.08 ± 3.01pg/mL) compared to HC subjects (31.3 ± 22.4 pg/mL) was revealed. Contrarily, the results showed a significant increase in the α-synuclein oligomers levels in PD patients (1.062 ± 0.266 ng/mL) compared to HC subjects (0.498 ± 0.203 ng/mL). As an added value to the previous study, these data showed that the total α-synuclein/oligomeric α-synuclein ratio can be quantified in salivary samples [40].

Shaheen et al. quantified the total and oligomeric forms of α-synuclein salivary in a 25 PD cohort with a different pathological score of disease compared to 15 HC subjects. Additionally, the authors correlated the total α-synuclein levels with disease severity. In line with the previous results, these data demonstrated a reduction of total α-synuclein (159.4 ± 61.6 ng/mL vs 229.9 ± 64 ng/mL) in salivary samples. Consequently, an increase of the total α-synuclein/oligomeric α-synuclein ratio was observed in PD patients (0.35 ± 0.18 ng/mL) compared to HC subjects (0.19 ± 0.08 ng/mL), but no correlation between the total α-synuclein levels and disease severity (*p*-value > 0.05) was found. In conclusion, this study confirmed the hypothesis of evaluating the total α-synuclein/oligomeric α-synuclein ratio as a possible biomarker in the diagnosis of PD. However, this ratio did not correlate with the severity of PD [41].

Cao et al. conducted a study aimed at the quantification of total α-synuclein and oligomeric α-synuclein in extracellular vesicles obtained from the saliva of 74 PD patients and 60 HC subjects. Western blot and Nanosight 300 were used to confirm the presence of extracellular vesicles in saliva. Additionally, the authors, by using electrochemiluminescence immunoassays, reported an increase in the oligomeric α-synuclein levels in the extracellular vesicles of PD patients (10.39 ± 1.46 pg/ng) compared to HC subjects (1.37 ± 0.24 pg/ng). Consequently, the total α-synuclein/oligomeric α-synuclein ratio in the extracellular vesicles increased in PD patients (1.70 ± 0.52 pg/ng) compared to HC subjects (0.67 ± 0.26 pg/ng). In conclusion, the authors showed that in extracellular vesicles derived from saliva, it is possible to quantify the total α-synuclein/oligomeric α-synuclein ratio [42].

Vivacqua et al. quantified the levels of oligomeric α-synuclein and total α-synuclein, as well as the total α-synuclein/oligomeric α-synuclein ratio in the saliva samples of 100 PD patients, 80 HC subjects, and 20 patients with progressive supranuclear palsy (PSP). The authors, using an ELISA kit, reported a decrease in total α-synuclein in the saliva of PD patients (7.104 ± 5.122 pg/mL) compared to HC subjects (29.091 ± 18.677 pg/mL). However, a significant increase of the salivary oligomeric α-synuclein was observed in PD patients (0.893 ± 1.949 ng/mL) compared to HC subjects (0.217 ± 0.191 ng/mL). Consequently, an increase in the total α-synuclein/oligomeric α-synuclein ratio was reported in PD patients (0.235 ± 0.793) compared to HC subjects (0.0126 ± 0.0079). In contrast, PSP patients showed no change in the concentration of total α-synuclein compared to HC subjects. Therefore, the results of this study demonstrated that the detection of α-synuclein in saliva could be used as a promising and easily accessible biomarker for PD but not for a differential diagnosis between PD and PSP [43].

The researchers also examined the DJ-1 protein, another PD-linked protein as a potential salivary biomarker in PD. Devic et al. quantified the levels of total α-synuclein and DJ-1 in the saliva of 24 PD patients and 25 HC subjects. The authors, using Western blot analysis, showed that total α-synuclein levels were lower in patients with PD compared to HC subjects. Conversely, in PD patients, a slight increase in salivary DJ-1 levels was detected. Additionally, the authors evaluated the link of these proteins with the severity of PD. The preliminary results of this study demonstrated that total α-synuclein and DJ-1 did not correlate to UPDRS motor scores. Therefore, these findings suggested that the DJ-1 protein in the saliva, as well as total α-synuclein, is correlated to PD. However, future studies will necessary to evaluate the potential utility of α-synuclein and DJ-1 as Parkinson’s disease biomarkers [44].

Stewart et al., using an immunohistochemical analysis, quantified the levels of total α-synuclein and DJ-1 in the cheek epithelium and saliva of 198 HC subjects compared to PD patients used in the previous study [44]. This study was aimed at evaluating how the age, sex of the subjects, or the origin of these proteins could influence their behavior. The results demonstrated that the concentration of α-synuclein and DJ-1 of HC subjects in cheek epithelium showed no difference compared to the cohort of PD patients. These results showed critical data on salivary changes in PD that should be considered in future investigations in order to evaluate salivary glands and saliva as a source of PD biomarkers [45].

Kang et al. conducted a study to evaluate the link between DJ-1 salivary concentrations and nigrostriatal dopaminergic function in order to monitor PD progression. The 74 PD patients and 12 HC subjects were subjected to photon emission tomographic examination with dopamine DATs (99mTc-TRODAT-1) in order to evaluate the brain function. The salivary DJ-1 levels were quantified using magnetic bead-based Luminex assays. A mild increase in DJ-1 in the saliva of PD patients (4.11 ± 5.88 ng/mL) compared to HC subjects (3.86 ± 5.44 ng/mL) was observed. However, in 74 PD patients, a slight correlation between DJ-1 salivary levels and putamen damage was found. Therefore, this study showed, for the first time, that DJ-1 could be used as a salivary biomarker for nigrostriatal dopaminergic function in PD [46].

Masters et al. quantified the concentration of total proteins, DJ-1, amylase, albumin, and mucins in the saliva of 16 PD patients and 22 HC subjects. The authors, using ELISA analysis, reported an increase in the concentration of total proteins (8.4 vs 5.0 mg/mL), amylase (127 vs 64 units/mL), and DJ-1 protein (0.84 vs 0.42 mg/mL) in the saliva of PD patients compared to HC subjects. Conversely, there was an increase in salivary albumin levels in PD patients (110 μg/mL) compared to HC subjects (47 μg/mL). No quantitative variation of the mucin was detected. The data obtained from this study suggested that saliva in PD patients showed a different composition than that of healthy subjects. These findings, thus, showed that saliva is a potential biological fluid to be used in the diagnosis of PD. However, the concomitant increase in DJ-1 and other salivary proteins in patients with PD may also indicate that salivary DJ-1 is not specific enough to serve as a biomarker in PD [47].

In conclusion, the results of these studies demonstrated that, using an ELISA kit, it is possible to quantify the levels of total and oligomeric a-synuclein in the saliva, thus showing that the ratio of total α-synuclein/oligomeric α-synuclein could be used as a biomarker for the diagnosis of PD. All these studies showed that there was no correlation between the amount of α-synuclein and the severity of the disease. It is likely that the high heterogeneity of the α-synuclein aggregates could be linked to specific stages of PD. However, the ELISA method used for quantification may not be able to detect all the subclasses of α-synuclein, so this determines a low sensitivity in differentiating patients with different severity PD.

The results of the quantifications of the DJ-1 protein in saliva showed changes in PD patients compared to healthy subjects. These data were in compliance with the results observed in the CSF.

### 3.2. Salivary Proteins Expressed Differentially: Potential Biomarkers in Parkinson’s Disease? 

Several studies have revealed that the saliva in PD patients has an abnormal composition compared to HC subjects. Consequently, researchers have evaluated how salivary proteins are differently expressed in PD patients and healthy subjects [48,49].

Song et al. quantified the heme oxygenase-1 (HO-1) in saliva samples of 58 patients with idiopathic PD and 59 HC subjects. This protein is involved in the etiopathogenesis of PD [49,50]. Though, in normal conditions, HO-1 showed neuroprotective effects [51], in PD patients, its increased level promotes the excessive accumulation of iron and carbon monoxide. These events induce an increase of oxidative stress and consequent cell damage [52]. The authors of this study, by using an ELISA kit and Western blot analysis, reported an increase in the saliva HO-1 levels in idiopathic PD patients (7.38 ± 95 ng/mL) compared to HC subjects (4.87 ± 0.68 ng/mL). Therefore, the increase of HO-1 concentrations in saliva demonstrated that it could be used as a potential biomarker for the diagnosis of early idiopathic PD [53].

Additionally, Costa et al. evaluated a relationship with the cortisol, the brain-derived neurotrophic factor (BDNF), and PD [54]. Some evidence has shown that in patients with PD, there may be a negative association with BDNF levels, motor deficits, and dopaminergic neuronal loss in the midbrain [55]. Meanwhile, cortisol, by suppressing both the synthesis and release of BDNF, may cause damage to the nigrostriatal system, with consequent dopaminergic degeneration [56,57]. Based on this evidence, Costa et al., using an ELISA kit, quantified the salivary cortisol and the levels of BDNF in the plasma of 18 PD patients compared to 17 HC subjects. An increase in salivary cortisol levels in PD patients (972.5 pg/mL) compared to HC subjects (425 pg/mL) was reported. Contrarily, no significant difference in plasma BDNF levels of PD patients (215.7 pg/mL) compared to HC subjects (340.1 pg/mL) was observed [54].

In Table 2, we summarize all the potential salivary biomarkers correlated with PD that were studied in the above-mentioned clinical studies.

## 4. Amyotrophic Lateral Sclerosis

Amyotrophic lateral sclerosis (ALS) is a neurological disease that leads to the degeneration of motor neurons. Therefore, this degeneration induces the gradual loss of control of various vital functions, such as walking, breathing, swallowing, and speaking [58,59]. The symptoms associated to ALS are muscle loss, cramps, and difficulty in speaking [60]. Furthermore, in patients with ALS, the loss of phrenic nerve function reduces diaphragm activity, which leads to orthopnea, dyspnoea, and hypoventilation. Indeed, the main cause of death in ALS patients is respiratory failure [59,60,61].

In 5–10% of patients, ASL has a genetic basis, while most cases are sporadic forms [62]. About 20% of familial ALS are characterized by mutations in superoxide dismutase-1 (*SOD1*), a gene that codes for the protein SOD1, which is involved in oxidative stress, with consequent neuronal death for apoptosis [62,63]. However, the causes of the ALS have not been fully elucidated. Environmental factors and lifestyle play an important role in the pathogenesis of ALS [64]: Eating habits, exposure to heavy metals or pesticides, alcohol, smoking, and a sedentary lifestyle can affect the onset of this disease [65].

Recently, researchers quantified salivary chromogranin (Cg) peptides in patients with ALS [66]. Chromogranin peptides such as chromogranin A (CgA), chromogranin B (CgB), and secretogranin II, are soluble neuroendocrine proteins contained within large dense-core vesicles, along with hormones and neuropeptides. The large dense-core vesicles, contained within neurons and cells of the neuroendocrine system, are responsible to the secretion of these molecules through exocytosis [66,67,68]. It has been demonstrated that CgA and CgB own motifs with a high affinity for the mutants SOD1. Within the vesicles, the Cg peptides bind to mutant SOD1. In this way, CgA and CgB, in a chaperone-like manner, mediate the selective secretion of misfolded SOD1 mutants. Therefore, the release of these SOD1 mutants mediated by CgA and CgB could be responsible for the activation of the microglia cells and the subsequent cell death of motor neurons. These findings would demonstrate the correlation between the peptides of Cg and ALS [69].

Recently, researchers quantified CgA, a soluble protein that plays an important role in the regulation of calcium and metabolism, in saliva [69,70]. Based on this evidence, Obayashi et al., using an ELISA kit, quantified CgA in the saliva samples of ALS patients with different scores of severity, in vascular dementia patients, and in HC subjects. The ALS patients, in the terminal phase, showed high salivary CgA levels (12.58 ± 2.79 pmol/mL) compared to patients with moderate ALS (6.36 ± 1.62 pmol/mL). In HC subjects, the concentration of CgA was low (3, 77 ± 1.90 pmol/mL), while patients with vascular dementia showed salivary CgA levels (4.04 ± 2.04 pmol/mL) similar to those of the HC subjects. A correlation between salivary CgA levels and the emotional state of ALS patients was observed. Therefore, these results encourage future studies to evaluate CgA as a possible salivary biomarker useful for the diagnosis of ALS [71].

## 5. Multiple Sclerosis

Multiple sclerosis (MS) is a demyelinating autoimmune disease that involves the central nervous system. The disease occurs more in females compared to males. MS is characterized by the infiltration of cells of the immune system, the activation of microglia, and progressive demyelination, which induces neuronal loss and consequent neurological damage [72]. The excessive immune-response causes the breakdown of the blood–brain barrier followed by the entry of T helper lymphocytes and B lymphocytes into the central nervous system. This cascade of events triggers the activation of an inflammatory reaction responsible for the demyelination process [73]. The symptoms associated with MS are pain, fatigue, visual problems, bowel and bladder dysfunction, depression, motor deficits, and sexual dysfunction [73,74]. MS shows several clinical variants, the most frequent being the relapsing–remitting MS (RR-MS), characterized by acute periods of neurological dysfunctions (relapsing) followed by periods of recovery (remitting) [75].

MS diagnosis is performed by MRI and CSF analysis. CSF analysis, performed by electrophoresis, aims for the detection of oligoclonal bands. The presence of oligoclonal bands indicates high quantities of immunoglobulin G (IgG) that reflect the excessive immune response in the central nervous system. The presence of IgG in the CSF can be expressed as the IgG index. The IgG index is an indicator of the relative amount of IgG in CSF normalized on serum albumin (expressed as a ratio of IgG in CSF/IgG in serum per albumin in serum/albumin in CSF). The result of the IgG index is elevated in about 70–90% of MS patients [76]. However, the oligoclonal bands are also present in other diseases, such as in some infectious, autoimmune pathologies and in cerebrovascular diseases. Therefore, the discovery of other easily accessible and specific biomarkers has raised the interest of the researchers [77]. Unlike CSF, saliva could be an accessible and non-invasive fluid for the identification of molecules useful for the diagnosis of MS.

### Salivary Biomarkers in Multiple Sclerosis

The increase in immunoglobulin A (IgA) in the CSF of MS patients, like the increase of IgG, could reflect an excessive immune response. IgA represents the main immune barrier against antigens in the mucous membranes and represents the predominant immunoglobulin in secretions such as saliva. Therefore, it was thought to research IgA in the saliva of patients with MS in order to evaluate if it could be used as a potential salivary marker for the diagnosis of MS [78,79].

Coyle et al., using ELISA kits, quantified the IgA levels in the saliva and tears of 21 MS patients and 19 HC subjects. A significant increase of monomeric IgA in 45% of saliva samples and in 56% of tears samples of MS patients was observed. The monomeric IgA was quantified only in two HC subject saliva samples. In conclusion, IgA was identified in the tears and saliva samples of MS patients. Therefore, the authors interpreted this data as indicative of the inflammatory state of the oral mucosa. The alteration of mucosal barriers could have implications in MS [78].

Pietz et al. quantified the levels of albumin, IgG, and IgA in the saliva of 11 MS patients treated with corticosteroids, 35 untreated MS patients, and 31 HC subjects. The authors reported albumin and IgG reductions in patients treated with corticosteroids compared to untreated MS patients and HC subjects. Conversely, treated MS patients observed no quantitative alterations in IgA. Instead, untreated MS patients showed a decrease in IgA compared to HC subjects. In conclusion, the dosage of IgA in saliva offers useful information to understand the state of the immune system of the disease [80].

Several studies have identified Ig free light chains (FLC), in monomeric and dimeric form, in the CSF of MS patients. The presence of FLCs in the CSF would demonstrate that the intrathecal production of these FLCs is high in MS [81,82,83].

In light of these findings, Kaplan et al., using Western blotting analysis, quantified the monomeric and dimeric FLCs in saliva samples of 58 RR-MS patients in the active phase of the disease, 15 RR-MS patients in the relapse phase, 12 secondary progressive MS (SP-MS) patients, and 28 HC subjects. The authors demonstrated that patients in the active phase of the disease (both RR-MS and SP-MS patients) showed an increase in FCL levels, compared to both patients with relapsing RR-MS and HC subjects. Conversely, the RR-MS in the relapsing phase showed levels of FLCs similar to the HC subjects. In conclusion, the salivary monomer–dimer FLC analysis could be used for MS diagnosis and to differentiate the relapsing phase versus the remitting phase [84].

Recently, an increase in class I and II human leukocyte antigen (HLA) molecules has been observed in several autoimmune diseases such as MS [85,86]. HLAs are coded to the major histocompatibility complex (MHC) gene cluster and are classified in HLA class I and HLA class II. HLAs play an important role, as they regulate the immune response following the presentation of the antigen through antigen-presenting cells (APCs). Consequently, the activation of dendritic cells, macrophages, astrocytes, and microglia occurs. Generally, HLAs are expressed in the surface of the cells responsible for the immune response. HLAs are also present in a soluble form (sHLA) in biological fluids, from which they can be quantified [87,88,89]. In MS, HLA-II regulates the immune response through the activation of cells directed against the myelin sheath. Indeed, several pieces of evidence have demonstrated elevated levels of sHLA-II in the serum and CSF of MS patients as a reflex of systemic immune activation [85,90].

Adamashvili et al., using an ELISA kit, quantified the levels of sHLA-I and sHLA-II in saliva and CSF samples of 13 RR-MS patients and 53 HC subjects. An increase in sHLA-II in the saliva and CSF samples of RR-MS patients compared to HC subjects was observed. It is noteworthy that in most samples, the levels of sHLA-II were similar in both saliva and CSF. Conversely, the sHLA-I was detected in none of the biological fluid. Increases in sHLA-I in saliva and CSF were only detected in two RR-MS patients in the relapsing phase. In conclusion, the results of the study demonstrated that the levels of sHLA-II showed a similar trend to that observed in the CSF. Furthermore, the possible correlation of sHLA to disease suggests that salivary sHLA could be used as a potential biomarker for the diagnosis of MS [89].

The same research team extended the study in order to assess the role of salivary sHLA-II in the therapeutic response to high-dose interferon beta-1a (IFN β-1a). The authors, using an ELISA kit, quantified sHLA-II levels in 17 patients with RR-MS at baseline and six months after treatment with IFN β-1a. The results of the study showed higher levels of sHLA-II in RR-MS patients at the baseline compared to 53 HC subjects. Six months after IFN β-1a treatment, sHLA-II levels were higher than at baseline. This finding highlighted a direct correlation between IFN β-1a and HLA-II. In conclusion, the results demonstrated that salivary sHLA-II could be used as a biomarker for the diagnosis of MS and to evaluate the therapeutic response to IFN β-1a [91].

Oxidative stress is one of the main factors involved in the etiopathogenesis of MS [92,93]. Indeed, several studies have demonstrated the role of oxidative stress in the pathogenesis of MS [94,95]. In line with this evidence, Karlik et al. quantified the levels of different markers of oxidative stress in saliva and serum samples from patients with MS in order to verify the potential of these biomarkers for the diagnosis of MS. Specifically, the authors, in order to evaluate the protein oxidation and lipoperoxidation, respectively, quantified advanced oxidation protein products (AOPP) and thiobarbituric acid reacting substances (TBARS). Additionally, in order to assessed carbonyl stress, advanced glycation end products (AGEs) and fructosamine were quantified. The results of the study showed that TBARS and AGE levels increased in the saliva of MS patients compared to HC subjects. Similarly, a significant increase of AOPP, TBARS, AGE and fructosamine levels was shown in the plasma of MS patients compared to HC subjects. Contrary, the AOPP remained unchanged in the saliva of MS patients. The results of this study showed the presence of high concentrations of oxidative stress markers in the saliva samples and serum of patients with MS, confirming the involvement of oxidative stress. These data are in line with the evidence present in the literature that recorded the presence of oxidative stress markers in plasma and CSF. However, oxidative stress markers are not specific, so future studies that support these data are needed [96].

## 6. Challenges and Limitations of Salivary Biomarkers

Saliva represents a new and accessible biological sample that can be collected non-invasively for the diagnosis of neurological diseases. Predominantly, salivary biomarkers are quantified using ELISA kits. Contrarily to the tests used for CSF analysis, the kits used for salivary biomarkers have not yet been standardized and validated for diagnostic use. However, it will be necessary to develop standardized tests for saliva and carry out studies that recruit larger population cohorts that allow for the identification of the exact cutoff values of salivary biomarkers.

In clinical practice, the Aβ_1–42_, t-TAU and p-TAU are biomarkers detected in the CSF of AD patients. These biomarkers can also be identified in the saliva sample of AD patients. However, the lack of the exact diagnostic concentration ranges limits the use of these salivary biomarkers in clinical analysis.

Currently, the diagnosis of PD is performed using clinical evaluation and neuroimaging, diagnostic means that do not appear to be effective enough for a diagnosis and to monitor the progression of the disease. Therefore, saliva could be an interesting biological fluid that is useful to diagnose PD and monitor its progression. The results of these studies have shown that two biomarkers can be identified in saliva: α-synuclein and DJ-1. Both of these could be possible salivary biomarkers for PD; however, it is necessary to develop standardized tests in order to obtain safe and reproducible results.

The observations reported for MS have shown that it is possible to dose IgG in saliva, and the quantified levels seem to follow the same trend observed in the CSF. Additionally, saliva samples also allow one to identify IgA, FLC, and HLA-II. However, the results of these data are still insufficient to classify these markers as possible indicators of pathology. Future studies will be needed to determine whether these markers are predictive of MS or are indicative of the presence of other immunological changes.

In conclusion, further studies are needed in order to implement the use of salivary biomarkers in clinical practice.

## 7. Conclusions

Studies performed in saliva have shown the possibility of quantifying biomarkers for the diagnosis of some neurodegenerative diseases such as AD, PD, and MS. Saliva represents an interesting fluid that can be easily collected and provides the possibility of having repeated samples. Therefore, several salivary biomarkers could be used for the diagnosis of neurodegenerative diseases. Regardless, the lack of standardization in the analysis method and the processing of the samples limits the use of saliva as a potential tool to employ in clinical analysis. However, the results obtained encourage researchers to investigate saliva so that it can be validated as a future biomarker for the early and less invasive diagnosis of neurodegenerative diseases.

## Figures and Tables

**Table 1:** Salivary biomarkers associated with Alzheimer’s disease (AD) described in clinical studies.

Biomarker	Biomaterial	Methods	Results	Sensitivity and Specificity of Biomarkers	N. Patients	References
Aβ_1–42_	saliva and plasma	ELISA	↑Aβ_1–42_ in AD patients (6.81 ± 20.04 pg/mL) vs PD patients (3.66 ± 4.21 pg/mL) vs HC subjects (2.89 ± 4.96 pg/mL)	Sensitivity and Specificity of around 90–95%	AD patients *n* = 70 mild AD patients *n* = 29 moderate AD patients *n* = 24 severe AD patients *n* = 17 PD patients *n* = 51 HC subject *n* = 56	[23]
			↑Aβ_1–42_ in mild AD patients (7.67 ± 16.25 pg/mL) vs severe AD patients (3.03 ± 3.49 pg/mL) vs moderate AD patients (11.70 ± 34.76)			
Aβ_1–42_	saliva	Antibody-based immunoassay with magnet nanoparticles	Significant ↑Aβ_1–42_ in AD patients compared to HC subjects	Higher sensitivity and higher specificity	AD patients *n* = 28 HC subject *n* = 17	[24]
Aβ_1–42_	saliva and tissue	ELISA	↑Aβ_1–42_ in AD patients (59.07 ± 6.33 pg/mL) vs HC subjects (22.06 ± 0.41 pg/mL)	Higher sensitivity and higher specificity	AD patients *n* = 10 HC subject *n* = 27	[22]
Aβ_1–42_	saliva	ELISA	Significant ↑ Aβ_1–42_ in AD patients (51.7 ± 1.6 pg/mL) vs HC subjects (21.1 ± 0.3 pg/mL)	Higher sensitivity and higher specificity	AD patients *n* = 15 HC subject n=8	[25]
Aβ_1–42_ and t-TAU/p-TAU ratio	saliva	Mass Spectrometry	Aβ_1–42_ was not detectable	Sensitivity of 99% and higher specificity of 95%	AD patients *n* = 21 HC subject *n* = 38	[26]
			Significant ↑ t-TAU/p-TAU ratio of AD patients (*p*-value <0.05) vs HC subjects			
Aβ_1–42_, p-TAU and t-TAU	saliva	ELISA	Aβ_1–42_ was not detectable	Low sensitivity	AD patients *n* = 20 PD patients *n* = 20 HC subject *n* = 20	[27]
			No significant difference in p-TAU and t-TAU in AD patients compared to PD patients compared to HC subjects Slight ↑ in p-TAU in AD patients compared to PD patients compared to HC subjects			
t-TAU/p-TAU ratio	saliva and CSF	Western blot analysis	↑ t-TAU/p-TAU ratio in the S396 site in the saliva of AD patients (*p*-value < 0.05) compared both to elderly healthy subjects and MCI patients No significant difference in t-TAU/p-TAU ratio in the CSF of AD patients compared to elderly healthy subjects	Sensitivity of 73% and specificity of 50%	AD patients *n* = 46 MCI patients *n* = 55 elderly healthy subjects *n* = 47	[28]
t-TAU	saliva	Ultra-sensitive single molecule array technology	No significant difference in t-TAU level in AD patients (12.3 ng/L) compared to MCI patients (9.8 ng/L) compared to elderly healthy subjects (9.6 ng/L)	Sensitivity of 91% and specificity of 100%	AD patients n=53 MCI patients *n* = 68 elderly healthy subjects *n* = 160	[29]

↑: increasing; ↓: decreasing; Aβ_1–42_: β_1–42_ amyloid peptide; Aβ_1–40_: β_1-40_ amyloid peptide; AD: Alzheimer’s disease; HC: healthy control; PD: Parkinson’s disease; t-TAU: total-TAU; p-TAU: phosphorylated-TAU; CSF: cerebrospinal fluid; MCI: mild cognitive impairment.

**Table 2:** Salivary biomarkers associated with Parkinson’s disease (PD) reported in clinical studies.

Biomarker	Biomaterial	Methods	Results	Sensitivity and Specificity of Biomarkers	N. Patients	References
total α-synuclein	saliva	ELISA	↓total α-synuclein in PD patients (65 ± 52.2 pg/mL) vs HC (314.01 ± 435.9 pg/mL)	High sensitivity and high specificity	PD patients *n* = 20 HC subject *n* = 20	[39]
total α-synuclein	saliva	ELISA	↓total α-synuclein in PD patients (5.08 ± 3.01 pg/mL) vs HC (31.3 ± 22.4 pg/mL)	Low sensitivity and low specificity	PD patients *n* = 60 HC subject *n* = 40	[40]
Oligomeric α-synuclein			↑oligmeric α-synuclein in PD patients (1.062 ± 0.266 ng/mL) vs HC (0.498 ± 0.203 ng/mL)			
Oligomeric α-synuclein/total α-synuclein ratio			↑oligomeric α-synuclein/total α-synuclein ratio in PD patients (0.174 ± 0.044) vs HC (0.065 ± 0.027)			
total α-synuclein	saliva	ELISA	↓total α-synuclein in PD patients (159.4 ± 61.6 ng/mL) vs HC (229.9 ± 64 ng/mL)	Sensitivity of 76% And specificity of 60%	PD patients *n* = 25 HC subject *n* = 15	[41]
α-synuclein oligomers			↑oligomeric α-synuclein in PD patients (47.8 ± 11.8 ng/mL) vs HC (39.2 ± 9.2 ng/mL)			
Oligomeric α-synuclein/total α-synuclein ratio			↑oligomeric α-synuclein/total α-synuclein ratio in PD patients (0.35 ± 0.18 ng/mL) vs HC (0.19 ± 0.08 ng/mL)			
Oligomeric α-synuclein	saliva	Electrochemiluminescence assays	↑Oligomeric α-synuclein in PD patients (10.39 ± 1.46 pg/ng) vs HC (1.37 ± 0.24 pg/ng)	Sensitivity of 92% and specificity of 86%	PD patients *n* = 74 HC subject *n* = 60	[42]
Oligomeric α-synuclein/ total α synuclein ratio			↑Oligomeric α-synuclein/total α-synuclein ratio in PD patients (1.70 ± 0.52 pg/ng) vs HC (0.67 ± 0.26 pg/ng)	Sensitivity of 81% and specificity of 71%		
total α-synuclein	saliva	ELISA	↓total α-synuclein in PD patients (7.104 ± 5.122 pg/mL) vs HC (29.091 ± 18.677 pg/mL)	Sensitivity of 67.44% and specificity of 91.04%	PD patients *n* = 100 HC subject *n* = 80	[43]
Oligomeric α-synuclein			↑oligomeric α-synuclein in PD patients (0.893 ± 1.949 ng/mL) vs HC (0.217 ± 0.191 ng/mL)	Sensitivity of 56.98% and specificity of 83.87%		
Oligomeric α-synuclein/total α-synuclein ratio			↑oligomeric α-synuclein/total α-synuclein ratio in PD patients (0.235 ± 0.793) vs HC (0.0126 ± 0.0079)	Sensitivity of 69.77% and specificity of 95.16%		
total α-synuclein	saliva	Western Blot	No significant difference total α-synuclein in PD patients vs HC	-	PD patients *n* = 24 HC subject *n* = 25	[44]
DJ-1			No significant difference DJ-1 in PD patients vs HC			
total α-synuclein and DJ-1	saliva and cheek epithelium	Immunohistochemical analysis	mild ↑total α –synuclein in females (0.45 ± 0.05 pg/µg) compared to males (0.34 ± 0.02 pg/µg)	-	HC subject *n* = 198 males subjects *n* = 137 females subject *n* = 61	[45]
			mild ↑ DJ-1 in males (179.8 ± 11.8 pg/µg) compared to females (194.8 ± 19.7 pg/µg)			
DJ-1 and 99mTc-TRODAT-1	saliva	Magnetic bead-based Luminex assays	↑ DJ-1 in PD patients (4.11 ± 5.88 ng/mL) vs HC (3.86 ± 5.44 ng/mL)	High sensitivity	PD patients *n* = 74 HC subject *n* = 12	[46]
			mild 99mTc-TRODAT-1 absorption in PD patients vs HC			
Total protein	saliva	ELISA	↑ total protein in PD patients (0.84 μg/mL) vs HC (0.42 μg/mL)	-	PD patients *n* = 16 HC subject *n* = 22	[47]
DJ-1			↑DJ-1 in PD patients (0.84 μg/mL) vs HC (0.42 μg/mL)			
amylase			↑amylase in PD patients (127 units/mL) vs HC (64 units/mL)			
albumin			↑albumin in PD patients (110 μg/mL) vs HC (47μg/mL)			
HO-1	saliva	ELISA	↑ HO-1 in idiopathic PD patients (7.38 ± 95ng/mL) vs HC (4.87 ± 0.68 ng/mL)	-	Idiopathic PD patients *n* = 58 HC subject *n* = 59	[53]
Cortisol	saliva	ELISA	↑ cortisol in PD patients (972.5 pg/mL) vs HC (425 pg/mL)	-	PD patients *n* = 18 HC subject *n* = 17	[54]
BDNF	plasma		NSS BDNF in PD patients (215.7 pg/mL) vs HC (340.1 pg/mL)			

↑: increasing; ↓: decreasing; α-synuclein: alpha-synuclein; PD: Parkinson’s disease; HC: healthy control; DJ-1: protein deglycase; HO-1: heme oxygenase-1; BDNF: brain-derived neurotrophic factor.
